# Optimal interval timing between transurethral resection of bladder tumors and Bacillus Calmette‐Guerin perfusion

**DOI:** 10.1002/cam4.6707

**Published:** 2023-11-14

**Authors:** Tao‐nong Cai, Jiang‐li Lu, Zhi Chen, Zhen‐hua Liu, Zhuo‐wei Liu, Kai Yao, Zi‐ke Qin, Yun‐lin Ye

**Affiliations:** ^1^ Department of Urology Sun Yat‐sen University Cancer Center Guangzhou China; ^2^ State Key Laboratory of Oncology in South China Sun Yat‐sen University Cancer Center Guangzhou China; ^3^ Collaborative Innovation Center for Cancer Medicine Guangzhou China; ^4^ Department of Hepatobiliary Surgery II Zhujiang Hospital of Southern Medical University Guangzhou China

**Keywords:** adverse reaction, BCG instillation, bladder cancer, interval time, TURBT

## Abstract

**Objective:**

There is no consensus regarding the best interval time between transurethral resection of a bladder tumor and Bacillus Calmette‐Guerin (BCG) perfusion. This study was to explore whether the interval time has an impact on the prognosis and adverse effects.

**Methods:**

We retrospectively reviewed the clinical data of patients who received BCG intravesical perfusion at Sun Yat‐sen University Cancer Center (SYSUCC) from September 2015 to October 2021. Recurrence‐free survival (RFS) and progression‐free survival were the primary endpoints. Cox regression was used to explore independent predictors. The association between interval time and adverse effect grade was detected by logistic regression. Propensity score matching (PSM) was performed.

**Results:**

A total of 403 patients were enrolled, the median interval time was 24 days (6–163 days), and the follow‐up was 28 months (7–82 months). Eighty‐eight (20.9%) patients relapsed, and 40 patients (10.0%) suffered progression. The multivariate Cox regression analysis confirmed that interval time was an independent predictor of RFS (*p* = 0.017). Notably, when the interval time was less than or equal to 26 days, there was a trend toward better RFS, PSM resulted in 65 matched pairs in each group, and Kaplan–Meier analysis showed that there was a significant difference in RFS between groups (*p* = 0.009). The logistic regression analysis showed that there was no correlation between interval time and adverse effects and their grades (*p* > 0.05).

**Conclusions:**

We considered that the first BCG perfusion could be performed within 2–4 weeks after surgery.

## Graphical abstract

At the present, intravesical Bacillus Calmette‐Guerin (BCG) instillation is one of the best therapies for treating non‐muscle‐invasive bladder cancer. But there is no consensus regarding the best interval time between transurethral resection of bladder tumor (TURBT) and BCG instillation. Moreover, there are no large sample data available that demonstrate whether the timing of primary instillation is associated with adverse outcomes. In our study, we found that the first instillation could be performed within 2‐4 weeks after surgery, and there is no correlation between the interval time and adverse reactions.

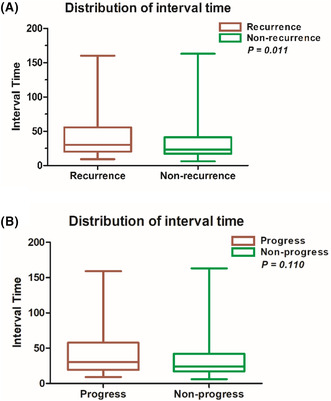

## 1 INTRODUCTION

Bladder cancer is one of the most common tumors of the urinary system.^1^ Based on myometrial invasion, bladder cancer can be divided into non‐muscle‐invasive bladder cancer (NMIBC) and muscle‐invasive bladder cancer (MIBC).^2^ To prevent the recurrence of tumors in the bladder, Bacillus Calmette‐Guerin (BCG) intravesical perfusion was recommended for intermediate‐risk or high‐risk NMIBC patients.^3^ However, to date, the optimal interval time between TURBT and BCG perfusion has been inconclusive. The European Association of Urology (EAU)^4^ states that initiating BCG at least 2 weeks post‐transurethral resection of the bladder, the National Comprehensive Cancer Network (NCCN)^5^ guidelines recommend within 3–4 weeks after surgery, and this issue was not even mentioned by the Canadian Urological Association (CUA) guideline.^6^ The optimal timing of administration remains unknown.

In addition, although the therapeutic effect of BCG was obvious, it simultaneously raised many adverse reactions during perfusion.^7^ Most patients present with gross hematuria and bladder irritation symptoms, and it cannot be ignored that BCG is a live attenuated strain of *Mycobacterium bovis* with bacterial properties that is known to invade the blood circulatory system through a wound or surgical injury and cause septicemia.^4^ Whether the interval time has an impact on adverse reactions during perfusion is also worthy of discussion. Therefore, to address these research gaps, we chose patients who received BCG intravesical perfusion after TURBT at the Sun Yat‐sen University Cancer Center (SYSUCC) as research cases and described our institution's experience.

## 2 PATIENTS AND METHODS

We retrospectively reviewed the clinical data of 403 patients who received intravesical BCG (D2PB302, China) perfusion after TURBT from September 2015 to October 2021 at the Sun Yat‐sen University Cancer Center (SYSUCC). A total of 120 mg of BCG was dissolved in 40–50 mL of saline and injected into the bladder via a urinary catheter. Patients excreted the drugs spontaneously after 2 h. During induction therapy, the drug was administered six times, once per week. If treatment was tolerated, patients were transitioned to the maintenance phase of treatment, and BCG was instilled once every 2 weeks for a total of 3 times, and then once every month until a year for a total of 19 treatments. Patients with high‐risk levels were dispensed one treatment per month for 2 years after the above treatment. Cystoscopy was reviewed every 3 months, and radiography was performed every 6 months within 2 years after surgery. Adverse effects were assessed by the Common Terminology Criteria for Adverse Events 5.0 (CTCAE 5.0). The prognostic evaluation was based on recurrence‐free survival (RFS) and progression‐free survival (PFS).

Statistical analyses were carried out using SPSS 20.0 software. Normally distributed variables are expressed as the mean ± standard deviation (SD), and nonnormally distributed variables are expressed as medians (interquartile range). The Chi‐square test was used for qualitative data, and the t test was used for quantitative data. The nonnormally distributed data were analyzed with the rank‐sum test. For paired samples, the Wilcoxon‐matched‐pairs test or McNemar test were utilized. The ROC curve was used to determine the cutoff value of interval time, and univariate and multivariate Cox regression were used to explore independent predictors. A propensity score‐matched analysis (PSM) was performed to rule out the influence of other factors, and Kaplan–Meier curve analysis was used to demonstrate the outcome of patients.

## 3 RESULTS

**FIGURE 1:**
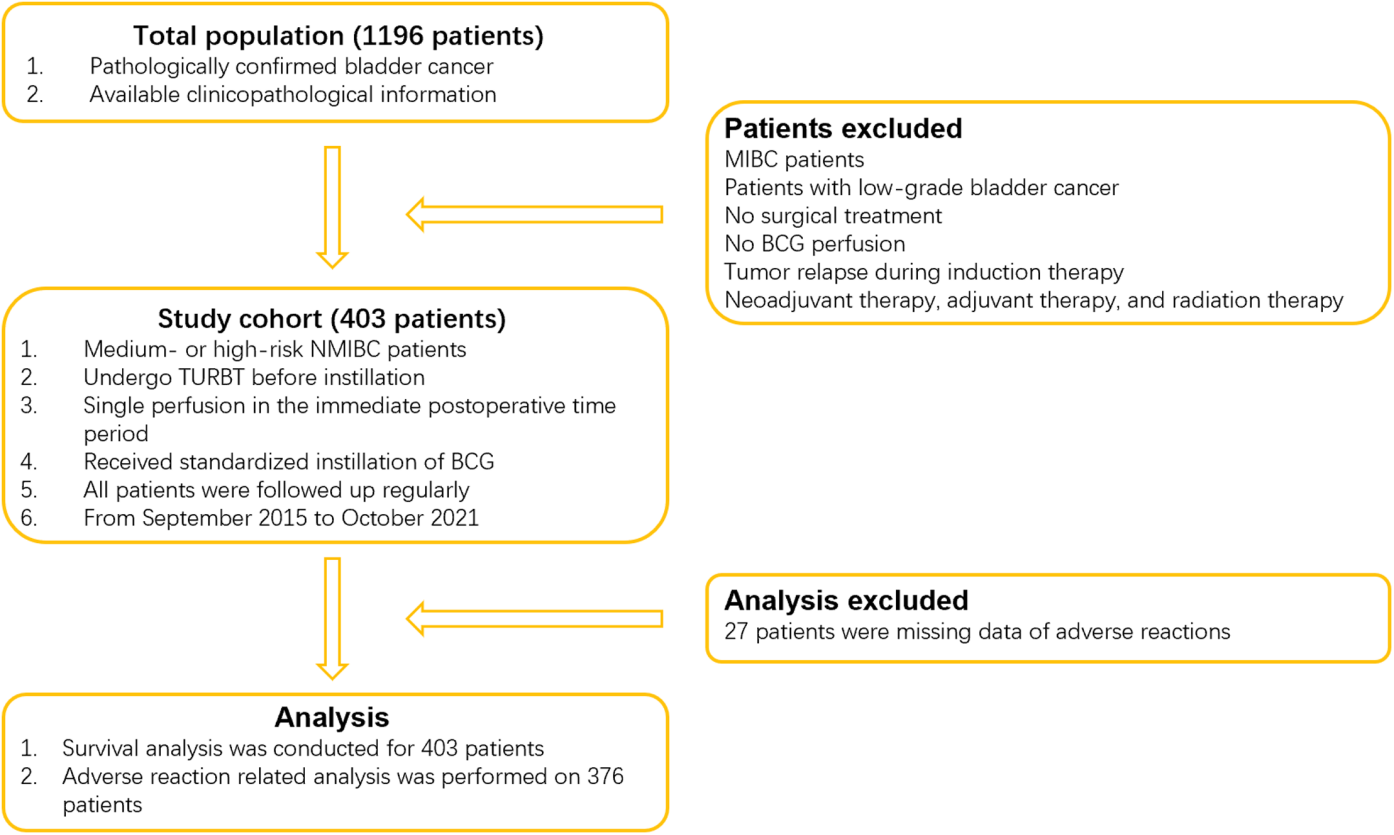
A total of 414 NMIBC patients were included in our study. All patients have received standardized instillation of BCG. Twenty‐seven patients were excluded owing to missing data of adverse reactions. In among, 403 patients were included in the research, and adverse reaction‐related analysis was performed on 376 patients. BCG: Bacille Calmette‐Guérin; MIBC: muscle‐invasive bladder cancer; NMIBC: non‐muscle‐invasive bladder cancer; TURBT: transurethral resection of bladder tumor.

**FIGURE 2:**
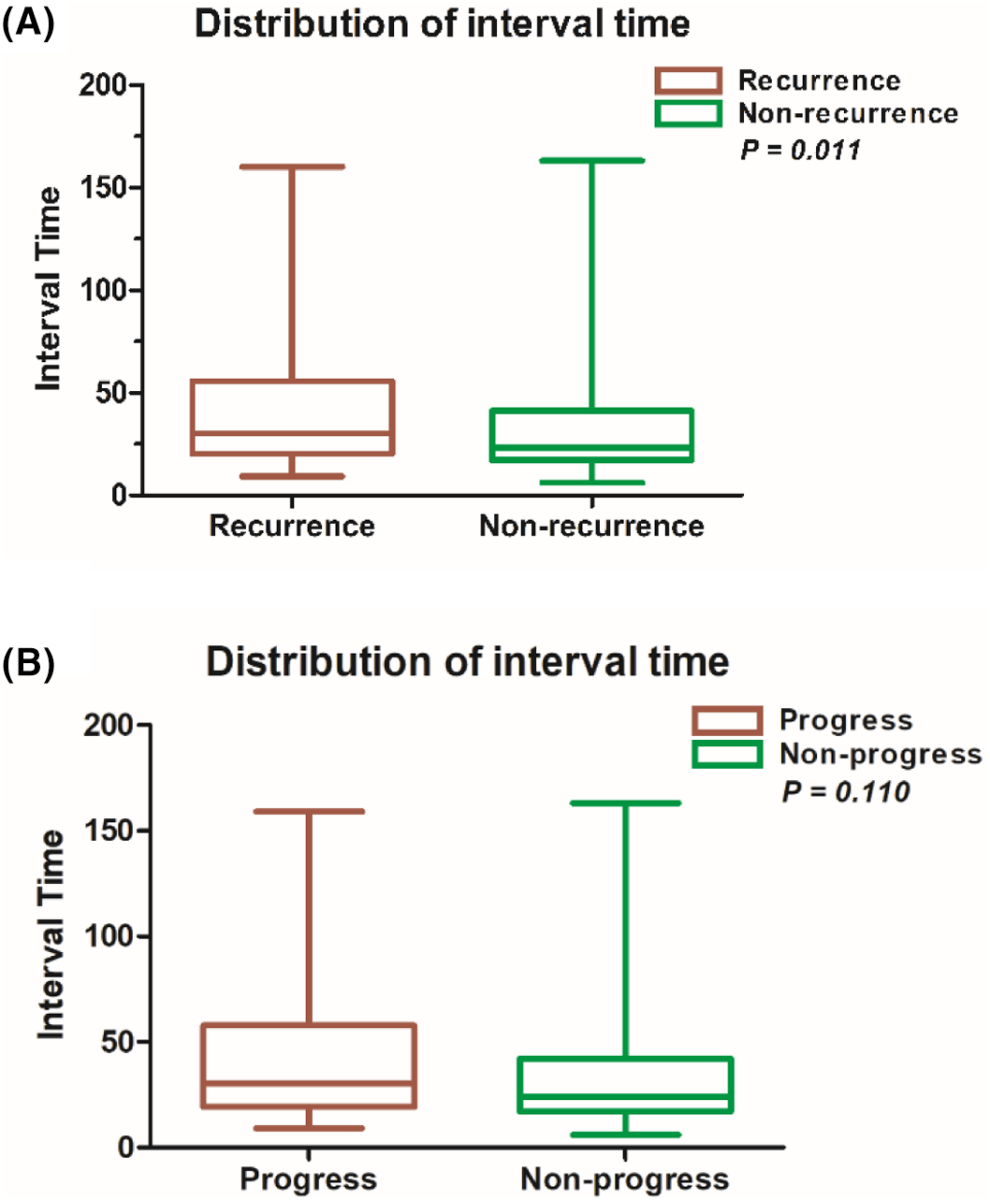
(A) Distribution of all patients' interval time; patients were divided into two groups according to whether they had recurrence; there were statistical differences between the two groups (*p* = 0.011); (B) Patients were divided into two groups according to progress; there were no statistical differences between the two groups (*p* = 0.110).

We retrospectively reviewed the clinical data of 403 patients who received BCG intravesical perfusion at Sun Yat‐sen University Cancer Center (SYSUCC) from September 2015 to October 2021 (Figure 1). The baseline characteristics of these patients are shown in Table 1. Notably, 187 patients had noninvasive bladder cancers (Ta or Tis), and 188 patients had invasive tumors (T1); the ratio was close to 1:1 (Figure 1). Patients were categorized into two groups depending on the interval time between TURBT and BCG perfusion, and recurrence differed significantly between the two groups (*p* = 0.011) (Figure 2A); there were no differences between the two groups when patients were grouped according to progress (*p* = 0.110) (Figure 2B). Adverse reactions were shown in 202 patients, of whom bladder irritation symptoms were the most common (60.9%). The time of first bladder tumor recurrence or progression was used as the primary endpoint for the study, and the median follow‐up was 28 months (7–82 months). Three patients had a follow‐up duration of less than 12 months, two died due to malignancies, and one was lost to follow‐up. During follow‐up, 88 patients suffered from tumor recurrence, and the median time to recurrence was 13 months (3–58 months). Forty patients suffered progression, and the median time was 19.5 months (4–51 months). Progression data were missing for 3 patients.

**TABLE 1:** Baseline characteristics of BCG instillation patients.

Variables	Value (*n* = 403)
Age (years)	62 (23–88)
Gender	
Male	327 (81.1%)
Female	76 (18.9%)
Tumor characteristics	
Initial	248 (64.9%)
Relapse	134 (35.1%)
Within 1 year	61 (46.2%)
Longer than 1 year	71 (53.8%)
Number of tumors	
Single	93 (29.1%)
Multiple	227 (70.9%)
Tumor size	
≤3 cm	236 (77.4%)
>3 cm	69 (22.6%)
T‐Stage	
pTa or pTis	187 (49.9%)
pT1	188 (50.1%)
Grade	
High	349(88.1%)
Low	47(11.9%)
Cis	
No	294 (95.5%)
Yes	14 (4.5%)
Muscularis propria present in pathological specimens	
Yes	229 (71.6%)
No	91 (28.4%)
Second resection	
Yes	121 (31.3%)
No	266 (68.7%)
Interval time between TURBT and BCG instillation (d)	24 (6–163)
Adverse reaction	
Grade 1	69 (34.2%)
Grade 2	110 (54.4%)
Grade 3	23 (11.4%)
Prognosis	
Recurrence	88 (21.8%)
Non‐recurrence	315 (78.2%)
Progression	40 (9.9%)
Non‐progression	363 (90.1%)

Abbreviations: BCG, bacille Calmette‐Guerin; CIS, concomitant carcinoma in situ; TURBT, transurethral resection of bladder tumor.

**FIGURE 3:**
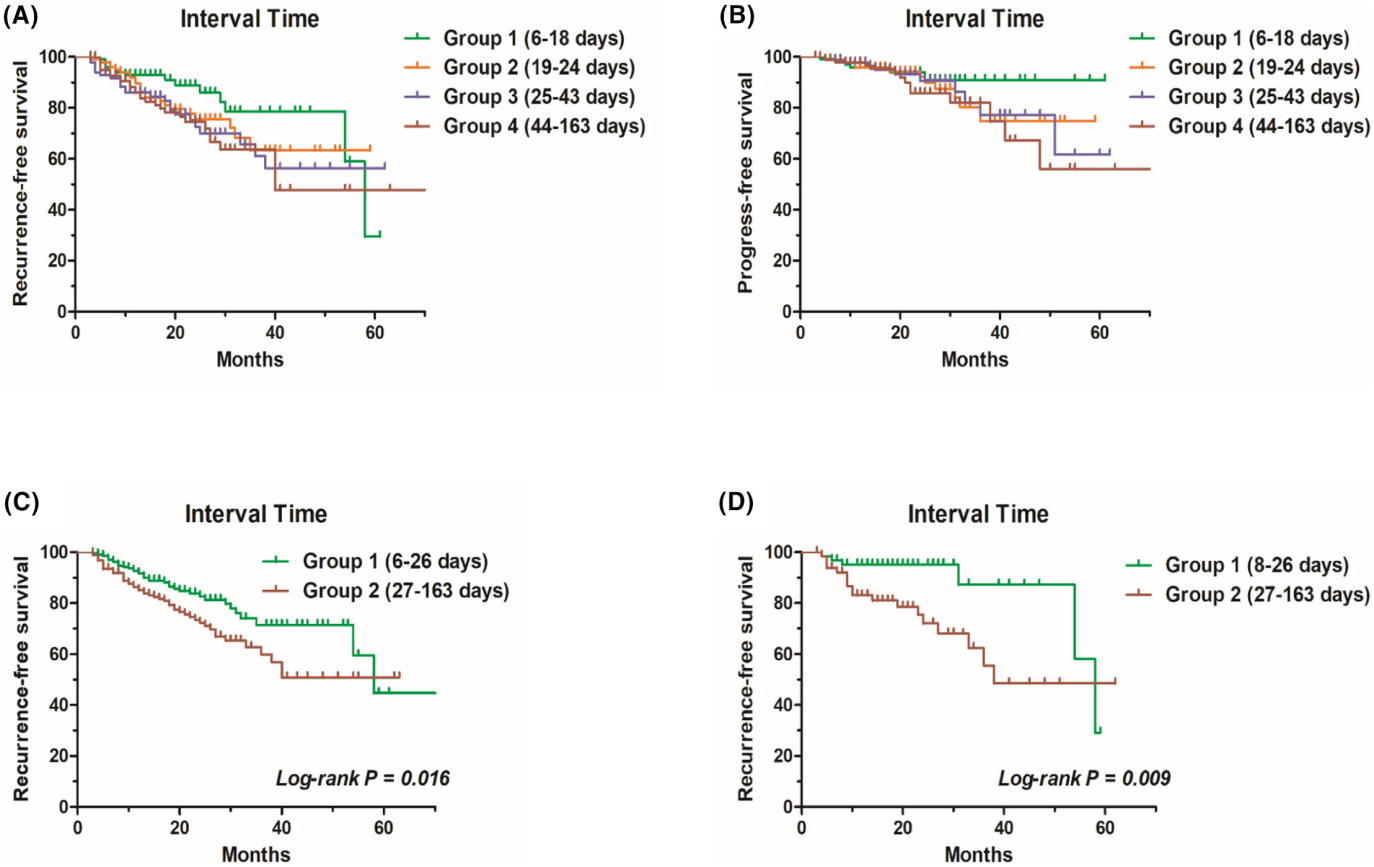
(A) Kaplan–Meier curve analysis of recurrence was done on all 403 patients, patients were stratified into four groups base on the quartiles, difference existed between Group 1 and 4 (*p* = 0.033), there were no differences between Group 1 and 2 (*p* = 0.128), Group 1 and 3 (*p* = 0.051), Group 2 and 3 (*p* = 0.583), Group 2 and 4 (*p* = 0.369), Group 3 and 4 (*p* = 0.739); (B) Kaplan–Meier curve analysis of progression was done, patients were stratified into four groups base on the quartiles, there were no differences between Group 1 and 2 (*p* = 0.322), Group 1 and 3 (*p* = 0.341), Group 1 and 4 (*p* = 0.140), Group 2 and 3 (*p* = 0.984), Group 2 and 4 (*p* = 0.489), Group 3 and 4 (*p* = 0.424); (C) Kaplan–Meier curve analysis was done on all patients, cutoff value was found by ROC curve, there was difference in the recurrence‐free survival between 2 groups (*p* = 0.016); (D) Kaplan–Meier curve analysis was performed for 65 matched pairs of patients after PSM, there were differences between 2 groups (*p* = 0.009).

We classified the interval time into 4 groups according to quartiles (6–18 days, 19–24 days, 25–43 days, and 44–163 days). The only significant difference in RFS was in Groups 1 and 4 (*p* = 0.033) (Figure 3A), and there was no significant difference in terms of progression among the 4 groups (Figure 3B).

The ROC curve was used to determine the cutoff of the interval time between TURBT and BCG perfusion, and patients were divided into two groups. When focusing on recurrence as an endpoint, the cutoff value of the interval time was 26.5 days, and when progression was the primary endpoint, the cutoff value was 56.5 days. The basic information, pathological data, and interval time of patients were incorporated into the Cox model for multivariate analysis (Tables 2 and 3). The results showed that time interval was an independent risk factor for recurrence but not for progression. Kaplan–Meier curve analysis revealed that the RFS between the two groups was significantly different (*p* = 0.016) (Figure 3C). Propensity score matching was performed to reduce selection bias between the two groups classified by interval time. Propensity score matching was performed using a multivariable logistic regression model based on age, sex, initial or relapse, time to relapse, number of tumors, T‐stage, grade, concomitant carcinoma in situ (CIS), tumor size, muscular propria present in pathological specimens, and second resection. Pairs of patients' interval times less than or equal to 26 days or higher than or equal to 27 days were derived using 1:1 greedy nearest neighbor matching within a PS score of 0.02. This strategy resulted in 65 matched pairs in each group (Table 4). Survival analysis was performed with Kaplan–Meier curve analysis, and the results showed that there was a significant difference in RFS between groups (*p* = 0.009), and the prognosis was more favorable in the group with an interval time less than or equal to 26 days (Figure 3D).

**TABLE 2:** Univariate analysis for risk factors for RFS after intravesical BCG therapy.

	RFS			
	Univariate		Multivariate	
Variable	HR (95% CI)	*p*‐Value	HR (95% CI)	*p*‐Value
Age (≤70 vs. >70 years)	1.727 (1.078–2.767)	**0.023**	1.517 (0.868–2.651)	0.143
Gender (Male vs. female)	0.828 (0.474–1.445)	0.506		
Tumorigenesis (Initial vs. relapse)	2.295 (1.494–3.526)	**<0.001**	2.383 (1.302–4.364)	**0.005**
Time to relapse (Within 1 year vs. more than 1 year)	0.598 (0.364–0.980)	**0.041**	0.844 (0.431–1.654)	0.622
Number of tumors (Single vs. multiple)	2.215 (1.191–4.118)	**0.012**	2.012 (1.040–3.892)	**0.038**
Tumor size (≤3 cm vs. >3 cm)	0.625 (0.318–1.228)	0.173		
T‐Stage (Ta or Tis vs. T1)	1.482 (0.948–2.316)	0.084	1.855 (1.123–3.063)	**0.016**
Grade (High vs. low)	1.103 (0.570–2.133)	0.772		
CIS (With vs. without)	1.597 (0.389–6.562)	0.516		
Muscularis propria present in pathological specimens (With vs. without)	0.724 (0.423–1.238)	0.238		
Second resection (With vs. without)	0.714 (0.463–1.102)	0.128		
Time interval between the initial section and instillation (≤26.5 vs. >26.5 days)	1.670 (1.094–2.551)	**0.018**	1.812 (1.113–2.951)	**0.017**

*Note*: *p* < 0.05, Bold values refered to significant difference in statistical analysis.

Abbreviation: RFS, recurrence‐free survival.

**TABLE 3:** Univariate analysis for risk factors for PFS after intravesical BCG therapy.

	PFS			
	Univariate		Multivariate	
Variable	HR (95% CI)	*p*‐Value	HR (95% CI)	*p*‐Value
Age (≤70 vs. >70 years)	2.236 (1.151–4.343)	**0.018**	2.133 (1.075–4.233)	**0.030**
Gender (Male vs. female)	0.593 (0.232–1.516)	0.275		
Tumorigenesis (Initial vs. relapse)	3.735 (1.919–7.272)	**<0.001**	3.662 (1.684–7.962)	**0.001**
Time to relapse (Within 1 year vs. more than 1 year)	0.464 (0.238–0.905)	**0.024**	0.934 (0.419–2.085)	0.868
Number of tumors (Single vs. multiple)	1.601(0.694–3.692)	0.270		
Tumor size (≤3 cm vs. >3 cm)	0.495 (0.172–1.425)	0.193		
T‐Stage (Ta or Tis vs. T1)	1.398 (0.728–2.683)	0.314		
Grade (High vs. low)	1.183 (0.463–3.021)	0.726		
CIS (With vs. without)	0.614 (0.146–2.582)	0.505		
Muscularis propria present in pathological specimens (With vs. without)	0.801 (0.372–1.726)	0.571		
Second resection(With vs. without)	0.634 (0.335–1.200)	0.161		
Time interval between the initial section and instillation (≤56.5 vs. >56.5 days)	1.907 (0.952–3.821)	0.069	1.988 (0.977–4.402)	0.058

*Note*: *p* < 0.05, Bold values refered to significant difference in statistical analysis.

Abbreviation: PFS, progress‐free survival.

**TABLE 4:** The baseline data of patients after PSM.

Characteristic	Group 1	Group 2	*p*‐Value
	8–26 days (*n* = 65)	27–163 days (*n* = 65)	
Age (Years)	65 (42–86)	64 (33–88)	0.530
Gender			0.077
Male	48 (73.8%)	56 (86.2%)	
Female	17 (26.2%)	9 (13.8%)	
Tumor characteristics			0.572
Initial	42 (64.6%)	46 (70.8%)	
Relapse	23(35.4%)	19 (29.2%)	1.000
Within 1 year	10 (43.5%)	9 (47.4%)	
More than 1 year	13 (56.5%)	10 (52.6%)	
Number of tumors			0.572
Single	19 (29.2%)	23 (35.4%)	
Multiple	46 (70.8%)	42 (64.6%)	
T‐Stage			0.868
pTa or pTis	33 (50.8%)	35 (53.8%)	
pT1	32 (49.2%)	30 (46.2%)	
Grade			0.424
High	58 (89.2%)	54 (83.1%)	
Low	7 (10.8%)	11 (16.9%)	
CIS			1.000
Yes	4 (6.2%)	3 (4.6%)	
No	61 (93.8%)	62 (95.4%)	
Tumor size (cm)	2.5 (0.5–6)	2.5 (0.5–6)	0.367
Muscularis propria present in pathological specimens			0.845
Yes	44 (67.7%)	46 70.8%)	
No	21 (32.3%)	19 (29.2%)	
Second resection			0.250
Yes	10 (15.4%)	7 (10.8%)	
No	55 (84.6%)	58 (89.2%)	
Duration of follow‐up (months)	17 (3–59)	18 (3–62)	0.941
Prognosis			**0.008** *
Recurrence	6 (9.2%)	18 (27.7%)	
Non‐recurrence	59 (90.8%)	47 (72.3%)	

* *p*‐value ≤ 0.05 was considered significant between the two groups.

Bold values refered to significant difference in statistical analysis.

Abbreviation: PSM, propensity score‐matched analysis.

Adverse effects data for 376 patients were included in the analysis; no adverse effects were observed in 174 patients (43.2%), 202 patients (56.8%) experienced adverse effects at follow‐up, 69 (34.2%) had grade 1 adverse reactions, 110 (54.4%) had grade 2 adverse reactions, and 23 (11.4%) had grade 3 adverse reactions. These 23 patients had to suspend or terminate BCG perfusion because of adverse reactions; 13 patients had to suspend or terminate BCG perfusion because the medication could not relieve hematuria and bladder irritation symptoms, and 10 cases had other causes (such as hyperpyrexia and lower abdomen distension). It is noteworthy that the interval time of 37 patients was less than or equal to 2 weeks, 26 patients (70.3%) reported adverse effects, and of the 339 patients greater than 2 weeks, 176 patients (51.9%) experienced adverse effects. From the data presented here, patients with an interval time less than 2 weeks were more prone to experience adverse reactions; the differences were statistically significant (*p* = 0.034), and the distribution of adverse effects was similar (*p* = 0.693) (Table 5).

**TABLE 5:** Adverse reactions of BCG instillation patients.

Variables	Number of patients	Adverse effects patients (%)	*p*‐Value	Grade (%)			*p*‐Value
				I	II	III	
All patients	376	202 (56.8)		69 (34.2)	110 (54.4)	23 (11.4)	
≤2 weeks	37	26 (70.3)	0.034	8 (30.8)	16 (61.5)	2 (7.7)	0.693
>2 weeks	339	176 (51.9)		61 (34.7)	94 (53.4)	21 (11.9)	

## 4 DISCUSSION

Bacillus Calmette‐Guerin is the first choice in intravesical perfusion treatment for intermediate‐risk or high‐risk NMIBC.^8^, ^9^ At present, advances have been achieved in perfusion times, perfusion period, and mechanism of action.^10^, ^11^, ^12^, ^13^ However, the timing of the initial perfusion is still controversial, and uniform treatment guidelines are also lacking.^14^ Moreover, there are no large‐sample data available that demonstrate whether the timing of primary perfusion is associated with adverse outcomes, which is still a matter of discussion.^15^ Herein, we found that patients tended to have better outcomes when the interval time was less than 4 weeks and fewer adverse reactions when the interval time was more than 2 weeks. To the best of our knowledge, the present study is the first to draw firm conclusions about the best timing of BCG primary perfusion, and the data for SYSUCC support our views.

In a recent study (Can Urol Assoc J, 2021, 15(8):230–239),^6^, ^16^ researchers collected the interval times of 518 patients, with a median time of 26 days (6–188 days). The patients were divided into 4 groups by quartile, and the results of survival analyses demonstrated that the differences in prognosis were statistically insignificant between these 4 groups. However, the optimum cutoff value was not determined in this study, and there were significant differences in baseline clinical data between the four groups, perhaps because other factors (such as T‐stage) affected the prognosis of patients. Therefore, in our studies, we divided categorized patients into two groups bounded by the interval time of 26 days (optimal cutoff value), and PSM was used to match patients in these two groups. The results suggested that the prognosis is more favorable when the interval time is less than or equal to 26 days. In addition, multivariate Cox analysis was performed on all 403 patients, and the interval time was an independent risk factor for recurrence. The present study shows that the first perfusion might be performed within 4 weeks after surgery, and patients tended to have longer relapse‐free intervals and progression‐free intervals.

Compared with other intravesical chemotherapy agents, BCG is more prone to adverse effects.^17^ Its complications include bladder irritation symptoms or hematuria, and the most serious and potential complication is BCG sepsis.^18^, ^19^ The EAU states that BCG perfusion should be performed 2 weeks after the end of surgery to avoid these severe adverse reactions.^4^ This may be because the bladder wall needs several weeks for a TURBT defect to re‐epithelialize and not allow BCG to be absorbed into the blood through the surgical incision, leading to BCG sepsis. However, 2 weeks seems to be the conventional interval time, and we could not find any proof of this in the literature. At the same time, because the incidence of BCG sepsis in patients is extremely low, such data are difficult to accurately collect. There has never been a conclusive study in support of an interval time greater than 2 weeks associated with a lower incidence of sepsis. In our data, we found that patients who underwent infusion 2 weeks after TURBT had a lower incidence of adverse reactions than those who underwent infusion within 2 weeks.

This is the first report of a Chinese‐made BCG relationship between interval time and various factors among Chinese people. A few limitations existed with the study. This is a single‐center retrospective study, and further work is required for validation by well‐designed, prospective, multicenter studies. However, the present study fills the gap in the guidelines and is representative of China.

## 5 CONCLUSION

The interval time between TURBT surgery and intravesical BCG perfusion was associated with the prognosis of high‐risk NMIBC, and the prognosis was more favorable in the group with an interval time less than or equal to 26 days. Moreover, an interval time of less than 2 weeks was more prone to be associated with adverse reactions. Therefore, we suggest that the first perfusion could be performed within 2–4 weeks after surgery.

## AUTHOR CONTRIBUTIONS

**Tao‐nong Cai:** Data curation (equal); project administration (equal); writing – original draft (equal); writing – review and editing (equal). **Jiang‐li Lu:** Data curation (equal); formal analysis (equal). **Zhi Chen:** Methodology (equal). **Zhen‐hua Liu:** Formal analysis (equal); supervision (equal). **Zhuo‐wei Liu:** Methodology (equal); supervision (equal); writing – original draft (equal); writing – review and editing (equal). **Kai Yao:** Methodology (equal); writing – original draft (equal); writing – review and editing (equal). **Zi‐ke Qin:** Project administration (equal); writing – original draft (equal); writing – review and editing (equal). **Yun‐lin Ye:** Project administration (equal); supervision (equal); writing – original draft (equal); writing – review and editing (equal).

## FUNDING INFORMATION

Guangdong Basic and Applied Basic Research Foundation (2022A1515111119).

## CONFLICT OF INTEREST STATEMENT

The author declares that there is no conflict of interest.

## ETHICS STATEMENT

This study was approved by the Sun Yat‐sen University Cancer Center ethics committee. (GZR2018‐053).

## RESEARCH INVOLVING HUMAN PARTICIPANTS AND/OR ANIMALS

This research is a retrospective study and involves research in humans.

## Data Availability

The data that support the findings of this study are available on request from the corresponding author. The data are not publicly available due to privacy or ethical restrictions.
